# The proportion of cancer-related entries in PubMed has increased considerably; is cancer truly “The Emperor of All Maladies”?

**DOI:** 10.1371/journal.pone.0173671

**Published:** 2017-03-10

**Authors:** Constantino Carlos Reyes-Aldasoro

**Affiliations:** School of Mathematics, Computer Science and Engineering, City, University of London, London, United Kingdom; Universita degli Studi di Roma Tor Vergata, ITALY

## Abstract

In this work, the public database of biomedical literature *PubMed* was mined using queries with combinations of keywords and year restrictions. It was found that the proportion of Cancer-related entries per year in PubMed has risen from around 6% in 1950 to more than 16% in 2016. This increase is not shared by other conditions such as AIDS, Malaria, Tuberculosis, Diabetes, Cardiovascular, Stroke and Infection some of which have, on the contrary, decreased as a proportion of the total entries per year. Organ-related queries were performed to analyse the variation of some specific cancers. A series of queries related to incidence, funding, and relationship with DNA, Computing and Mathematics, were performed to test correlation between the keywords, with the hope of elucidating the cause behind the rise of Cancer in PubMed. Interestingly, the proportion of Cancer-related entries that contain “DNA”, “Computational” or “Mathematical” have increased, which suggests that the impact of these scientific advances on Cancer has been stronger than in other conditions. It is important to highlight that the results obtained with the data mining approach here presented are limited to the presence or absence of the keywords on a single, yet extensive, database. Therefore, results should be observed with caution. All the data used for this work is publicly available through PubMed and the UK’s Office for National Statistics. All queries and figures were generated with the software platform Matlab and the files are available as supplementary material.

## Introduction

The database *MEDLINE* of the United States National Library of Medicine (NLM) and its search engine *PubMed* (https://www.ncbi.nlm.nih.gov/pubmed) have grown to include over 26 million entries: 26,710,394 on the 30 November 2016. In MEDLINE, each entry is indexed with Medical Subject Headings (MeSH) and various field descriptors such as author, date, title, publication type, etc. For a complete list of the MEDLINE elements, the reader is referred to https://www.nlm.nih.gov/bsd/mms/medlineelements.html. These fields allow specific searches to be performed in PubMed by restricting the search to one, several or all fields and logical combinations with operators such as AND, OR, NOT are available as well. Whilst PubMed it is not without criticism for its inconsistency of terminology [1,2], document ranking not content-based [3], and “unwelcoming complexity” of its searches and terms [4], PubMed is considered to be “the most widely used database of biomedical literature” [3] and it was found to have better precision than Google Scholar (http://scholar.google.com) [5]. An interesting discussion of advantages and disadvantages of PubMed is summarised in a website titled “*Twenty million papers in PubMed*: *a triumph or a tragedy*?” [6].

This work was motivated by the interest on the presence of Cancer-related publications in PubMed. Within the database of PubMed, more than 3 million entries correspond to Cancer, which correspond roughly to 12% of the total entries. However, the proportion has increased significantly from around 6% in the 1950 to 16% in 2016. To investigate this increase and its possible causes, this work generated data mining tools to perform a systematic mining of Cancer-related terms in PubMed. The platform selected for the mining was Matlab^®^ (The Mathworks ^™^, Natick, USA) which is a widely-used programming environment in Science and Engineering. All the code used for this publication is provided in S1 File. Matlab can be used to retrieve the information and further analyse or display the data with its powerful tools, without the need of specialised web-based tools like MeSHy [3] or GoPubMed [7]. Furthermore, data from other websites like the UK’s Office for National Statistics (https://www.ons.gov.uk) was also extracted and displayed with Matlab for this work. The results are essentially limited as they are restricted to one database and the presence or absence of the keywords used in this study.

## Materials and methods

MEDLINE PubMed was queried with a combination of keywords using the software platform Matlab^®^ (The Mathworks ^™^, Natick, USA) and displayed directly with Matlab. The keywords were combined to form a Uniform Resource Locator (URL), which started with the basic address of PubMed 'https://www.ncbi.nlm.nih.gov/pubmed/?term=', to which the keywords to be searched in ‘[All Fields]’ or ‘[MeSH Terms]’ were added. URLs do not accept special characters like spaces, brackets [] or quotes ' " and these need to be converted to the ASCII character set. For instance, the following PubMed search "Cancer"[All Fields] has to be translated to the following URL: https://www.ncbi.nlm.nih.gov/pubmed/?term=%22Cancer%22%5BAll+Fields%5D.

A series of hypothesis were investigated by adding keywords to the URL. Queries on the terminology related to Cancer were investigated with the keywords: *neoplasms*, *cancer*, *tumor*, *neoplasm*, *tumors*, *oncology*, *metastasis*, *cancers*, *tumour*, *tumours* and *neoplasia*. *The growth of Cancer was compared against other conditions*, *namely*: *AIDS*, *Malaria*, *Tuberculosis*, *Diabetes*, *Cardiovascular*, *Stroke* and *Infection*. *Queries for organ-related cancers were investigated with the keywords*: *Bladder*, *Bowel*, *Brain*, *Breast*, *Kidney*, *Leukaemia*, *Liver*, *Lung*, *Lymphoma*, *Melanoma*, *Mouth*, *Ovarian*, *Pancreas*, *Prostate*, *Sarcoma*, *Stomach*, *Testicular*, and *Uterus*. Year-on-year variation on Cancer and other conditions were queried by restricting the date of publication to a single year.

The URL was passed as an argument to the Matlab command ‘*urlread’*, which read and returned a variable with the webpage that PubMed produced for that specific query. The webpage was stored in a string variable as a sequence of alphanumeric characters. The variable was then searched for the string ‘count’ which provided the number of entries contained in that search. To restrict the search to one particular year, e.g. 1990, the following term was added to the search: ‘AND "1990"[DP]’ where ‘DP’ is the MEDLINE Field for ‘Date of Publication’. These yearly queries were used to perform searches between 1950 and 2016.

The code to generate the queries and the figures is included in S1 File. There were no restrictions of language, country or publication type. However, if necessary, it would be easy to use these MEDLINE field with the source code provided with this work.

## Results and discussion

### Terminology

**Fig 1:**
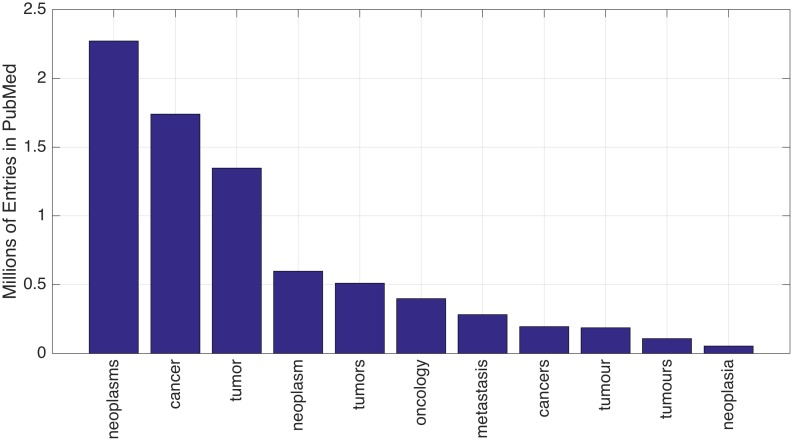
Number of cancer-related entries for different keywords listed in decreasing order.

To investigate the variability of terminology [1] and in particular of Cancer keywords [2], several keywords and some of their combinations were explored. The search indicated that the Cancer-related keyword with more entries was ‘*neoplasms’* with more than 2 million entries followed by *'cancer'*, *'tumor'*, *'neoplasm'*, *'tumors'*, *'oncology'*, *'metastasis'*, *'cancers'*, *'tumour'*, *'tumours'* and *'neoplasia'* (Fig 1). It was interesting to notice that whilst there are more entries for ‘*neoplasms’* in plural than for ‘*neoplasm’*, the opposite was true for ‘*tumor/tumors’*, ‘*cancer/cancers’* and ‘*tumour/tumours’* where the term in singular resulted in a larger number of entries. It would have been expected that a term in singular would include the plural, but for neoplasms this was not the case.

**Fig 2:**
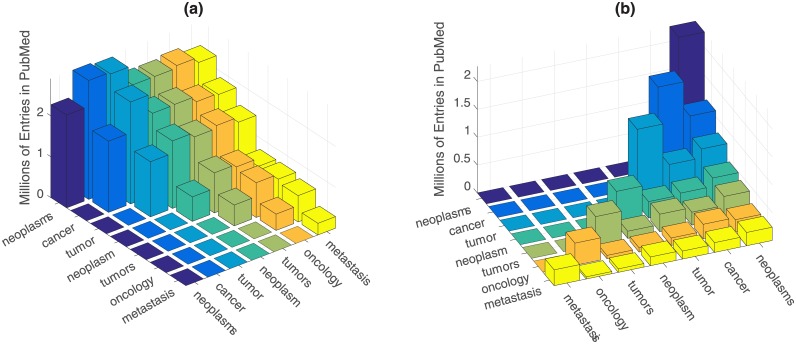
Number of entries in PubMed for searches with pairs of keywords. For all cases, each column represents the result for the combination of the pair of keyword on the two axes. (a) Combinations with the operator OR. (a) Combinations with the operator AND. The diagonal corresponds to a single keyword and since the matrix is symmetric a single side is shown.

The overlap of terms for a single entry was explored by performing pair-wise searches between all keywords: i.e. ‘*neoplasms* OR *cancer’*, ‘*neoplasms* OR *tumor’*, etc. Partial results are shown in Fig 2a as bars indicating the number of combined entries. Since these searches are symmetric, only one case is shown with the diagonal indicating the results with the single entry (i.e. *tumor* OR *tumor*). Whilst it was expected that combinations of distinct keywords would report an increase of entries with the combination (e.g. *neoplasms* = 2,271,532, *cancer* = 1,742,236, *neoplasms* OR *cancer* = 2,909,324) it was curious to notice that the combinations of singular and plural terms also increased the number of entries (e.g. *tumor* = 1,349,097, *tumors* = 510,353, *tumor* OR *tumors* = 1,543,897. These results indicated that there were entries, which used one, the other or both keywords.

The repetition of the keywords was explored by changing the term OR for AND, i.e. ‘*neoplasms* AND *cancer’*, ‘*neoplasms* AND *tumor’*. Partial results shown in Fig 2b show a considerable number of publications that contain both keywords. Again, the diagonal contains the result of a single keyword and only one combination is shown, as the search is symmetric. These results together those of Fig 2a indicate that single words are not sufficient to search for Cancer in PubMed. Another observation of these searches is that the number of entries with American spelling for *tumor* (1,347,989) was around sevenfold larger that its counterpart in British spelling *tumour* (187,719).

### Year to year PubMed entries of cancer and other conditions

**Fig 3:**
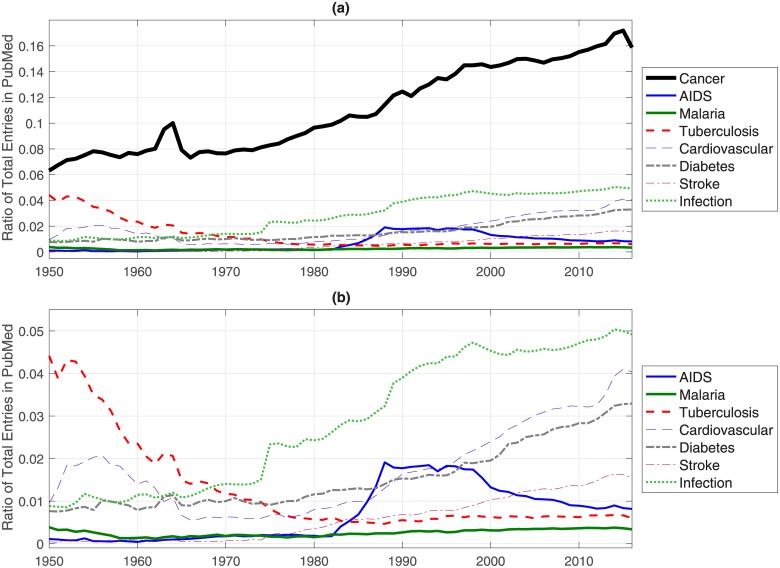
(a). Ratio of a series of condition-related entries in PubMed to the total number of entries per year. Notice how Cancer entries have increased from around 6% in the 1950s to 16% in 2016. All other conditions are considerably below Cancer. (b) Zoom into the lower values of the vertical axis of (a). Notice the different trends of each condition.

Next, year-to-year queries were performed for to investigate the number of PubMed entries that were indexed in a given year. The Cancer-related entries were as defined by a subset of the keywords previously mentioned (*neoplasms*, *cancer*, *tumor*, *tumour*, *oncology*) in all of the MEDLINE fields. These were compared against the total number of entries of a single year; in these cases, the query was restricted only to the date of publication. The proportion of Cancer-related entries to the total is shown in Fig 3a, where it can be seen a rise from 6% in 1950 to 16% in 2016. To compare this rise, the following conditions were also queried on a yearly basis: *AIDS*, *Malaria*, *Tuberculosis*, *Diabetes*, *Cardiovascular*, *Stroke* and the more general *Infection*. In the case of *AIDS*, the query was performed with the combination of the following keywords: "*acquired immunodeficiency syndrome*" [MeSH Terms] OR ("*acquired*" [All Fields] AND "*immunodeficiency*" [All Fields] AND "*syndrome*" [All Fields]) OR "*acquired immunodeficiency syndrome*" [All Fields] OR "*aids*" [All Fields]. The other conditions were sufficiently specific to use only one keyword. A zoom into the lower portion of the graph is shown in Fig 3b.

The trends shown in Fig 3 are quite interesting. First, none of the other conditions is close to Cancer in the number of entries in PubMed. Even the more general term Infection is more than 10% below in 2016. Second, the trend of growth of Cancer is way higher than any of the other conditions growing from 8% in the 1970 to 16% in 2016.

The proportion of AIDS-related entries rapidly increased from nearly zero to close to 2% in a few years in the 1980s. The peak in the late 1980s is close to the development of anti-retroviral treatments like *zidovudine* [8–10] and their approval, as a therapy against HIV, by the US Food and Drug Administration (FDA) (10,11) in 1987. The entries remained relatively constant until the late 1990s and after that date there has been a steady decline in the number of entries. According to a recent study, during this time the “effective number of infections” plateaued in the 1990s in the United States [11]. However, the deaths of HIV/AIDS were continued to rise to become one of the leading causes of death globally. According to Lozano [12], the fatalities caused by AIDS were 0.30 million in 1990, 1.5 million in 2010, and reached a peak of 1.7 million in 2006. Clearly, the number of entries indexed by PubMed, has diverted to other areas.

Even when *Cardiovascular* disease is considered “the world’s top killer” [13,14], the contribution in PubMed oscillates between 0.5% - 4%. Interestingly, it was close to 2% in the 1950s, then dropped and remained at a low level until the 1980s, from which it has grown steadily with a very recent sharp increase. The decreasing trend may be related to a decrease in mortality rate, probably due to the decrease on some risk factors like smoking, and high blood pressure. In the United States the rates of death from coronary heart disease peaked in 1968 [15]. The increase experienced since the 1990s may be related to the sharp increase in two other risk factors: obesity and diabetes [16].

The entries related to the term *Infection*, have risen from 1% in the 1950s to around 5% in 2016. Whilst this is a significant increase and a position higher than most other conditions queried in this work, it still does not correspond to ranking in worldwide mortality. According to World Health Organization [17,18] lower respiratory infections constitute the third leading cause of death and diarrhoeal diseases, most of which are infections of the intestines, are the sixth cause of death.

*Diabetes* and *stroke* have shown similar trends, from steady levels of 1% and near to 0%, respectively, between 1950 and 1980s, and then a steady increase to reach around 3% and 1.5% respectively, in 2016. The increase of research in both conditions may be related with what some reports consider “alarming increase” in obesity [15,19,20], as obesity together with sedentary lifestyles are associated with significant higher rates of diabetes [16,21,22] and obesity is a risk factor for stroke and one of its most common co-morbidities [23–26]. There were reports of an “obesity paradox” [27] where “in cardiovascular disease populations, obese patients survive better”, however, this has been proven to be result of a biased selection [28]. As with other conditions, the proportion of research entries does not reflect the rank of global causes of death [12] of these conditions.

An interesting case is related to *Tuberculosis* (TB). It has been reported that the absolute number of research entries related to Tuberculosis has increased [29], however, as a proportion to the total research reported in PubMed, it has decreased from around 4% in the 1950, when it was the second after Cancer (Fig 3), to a lowest point of 0.5% in the late 1980s and then experiencing a minor increase, probably related to the United States (US) Government National Tuberculosis Elimination plan released in 1989 [30]. Even when the number of cases of TB reported in the US is relatively small, 9,951 in 2012, as compared with the global 8.6 million cases and 1.3 million deaths [31], there is significant concern as the majority of the TB reported cases in the US correspond to individuals born outside the US, thus reducing TB overseas would reduce the domestic cases [32].

Despite its significant contribution of global causes of death [12], the ratio of entries related to *Malaria* is below 0.5% for the whole period of analysis. The reasons behind this low level must be numerous and complicated. One report observed that research investment in Malaria followed colonial ties between the United Kingdom and former colonies [33]. Sometimes, it is considered that investment in drug development, especially from the private sector, is dedicated exclusively to drugs that will be marketable and profitable in the developed world [34,35]. This has led to some diseases, like Malaria to be considered as “neglected” [36] and others like sleeping sickness and Chagas as “most neglected” [34]. There has been progress with funding from public-private initiatives, notably *The Global Fund to Fight AIDS*, *Tuberculosis*, *and Malaria* [37]. However, it has been observed that the funding for the control of Malaria is still inadequate [38] and that efficient prioritisation of resources could save greater number of lives [39].

Finally, the combination of all the previous terms accounted to 31.42% of the entries indexed on 2016. To investigate the remaining 68.58% a series of queries with keywords from the MESH terms were performed. The results are presented as percentages in brackets after the keyword: *Adrenal (0*.*23%)*, *Aggression (0*.*15%)*, *Alcohol (1*.*07%)*, *Allergy (0*.*79%)*, *Alzheimer's (0*.*68%)*, *Antigen (0*.*77%)*, *Behavior (3*.*74%)*, *Chemistry (8*.*69%)*, *Dementia (0*.*61%)*, *Enzyme (2*.*13%)*, *Epidemiology (4*.*74%)*, *Epilepsy (0*.*48%)*, *Fracture (0*.*75%)*, *Fungal (0*.*63%)*, *Geriatric (0*.*45%)*, *Glaucoma (0*.*23%)*, *Hepatitis (0*.*66%)*, *Hormone (0*.*87%)*, *Inheritance (0*.*13%)*, *Injections (0*.*51%)*, *Insurance (0*.*45%)*, *Labor (0*.*28%)*, *Lactose (0*.*06%)*, *Leprosy (0*.*05%)*, *Life Expectancy (0*.*11%)*, *Lupus (0*.*25%)*, *Macular Degeneration (0*.*12%)*, *Magnetic Resonance (1*.*95%)*, *Microscopy (2*.*05%)*, *Mitochondrial (1*.*03%)*, *Morgue (0%)*, *Neurology (2*.*63%)*, *Nutrition (2*.*11%)*, *Nursing (2*.*04%)*, *Osteoporosis (0*.*29%)*, *Parasitic (0*.*27%)*, *PCR (1*.*74%)*, *Plaque (0*.*34%)*, *Poisoning (0*.*18%)*, *Polymers (0*.*52%)*, *Phobia (0*.*03%)*, *Therapy (8*.*39%)*, *Tomography (1*.*92%)*, *Toxins (0*.*22%)*, *Vasectomy (0%)*, *Vitamin (0*.*66%)*, *Wound healing (0*.*33%)*. It should be noticed that only 2 of the extra 47 keywords were above 5%: “Chemistry” with 8.69% and “Therapy” at 8.39%. Another important observation is that there may be entries that are related to more than one keyword and would appear in more than one query. The total for these 47 keywords is 56.32%.

### Year-to-year PubMed entries of organ-specific cancer queries

**Fig 4:**
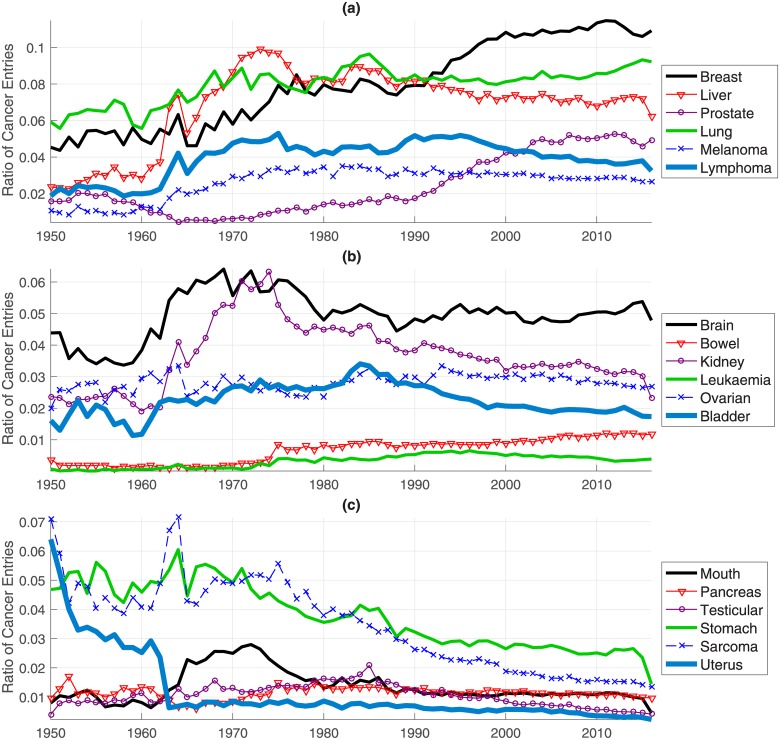
Ratios of the cancer entries related to organ-specific keywords. The trends have been ranked and presented according to (a) largest increase, (b) intermediate increase and (c) largest decrease from 1950s to 2016.

The year-to-year results can be further explored by using organ-specific keywords to investigate the trends related to some specific Cancers. The following 18 keywords were used to explore some common Cancers: *Bladder*, *Bowel*, *Brain*, *Breast*, *Kidney*, *Leukaemia*, *Liver*, *Lung*, *Lymphoma*, *Melanoma*, *Mouth*, *Ovarian*, *Pancreas*, *Prostate*, *Sarcoma*, *Stomach*, *Testicular*, *Uterus*. Furthermore, the results were ranked by the difference between the percentages in the periods 1950–1955 and 2011–2016 to investigate if the use of a specific keyword had increased or decreased. The results with the highest increase are shown in Fig 4a, the highest decrease in Fig 4c and intermediate results in Fig 4b. The highest increase was related to the keyword *Breast* from 4.5% to nearly 11% of the cases, followed by *Liver* and *Prostate*. The highest decrease was for the keyword *Uterus* from 6% to 0.2% followed by *Sarcoma* and *Stomach*. It is important to highlight that an increase or decrease in the number of entries does not necessarily imply an increase or decrease on the incidence of the organ-related Cancer; it may be that there was a change on terminology. This may be the case for *Uterus*, which experienced a dramatic drop between 1962 and 1963. Further investigation into that drop is beyond the scope of this publication.

### Why have cancer entries risen in this way in PubMed?

As an attempt to unravel the reasons behind the increase, which could be considered as dominance, of Cancer-related entries in PubMed, several hypotheses were examined.

The first hypothesis to be tested was the following: *Cancer incidence is increasing and thus the research related to Cancer has increased*. The literature has a considerable number of reports in which the incidence of certain Cancers has increased, and others for which the incidence has decreased. In some cases, the *decrease* in incidence has been related to a geographic population: cervical cancer in Spain [40], renal cell carcinoma in Sweden [41], colorectal cancer in the United States [42], testicular cancer in Denmark [43]. In other cases, the reduction has been linked with changes of treatments, notably hormone replacement therapy [44,45] or the use of screening or testing like Prostate Specific Antigen (PSA) for prostate cancer in Italy [46]. On the other hand, *increase* of cancer of the tongue has been recorded in Nordic countries [47], thyroid cancer in the U.S. [48] and Australia [49], distant stage breast cancer in the US [50] and India [51]. Variations in screening and lifestyle factors are considered to provide substantial incidence variation [52] but others suggest variations of other risk factors [53].

As an examination of this hypothesis, Cancer in the United Kingdom (UK), as a subset of the global population, was investigated in a similar way as the mining of the previous figures. The incidence and mortality data was mined, again with Matlab from the Office for National Statistics (http://www.ons.gov.uk/). Whilst the incidence of all reported Cancers in the UK has slightly increased between 1995 and 2014, the growth is nothing comparable to the rise shown in Fig 3. Furthermore, the trend in mortality has decreased in the same period.

**Fig 5:**
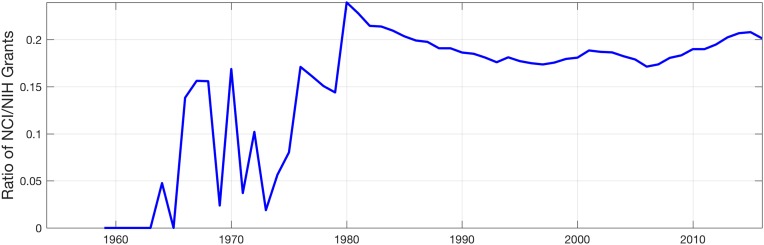
Ratio of the number of entries that report a grant number of the National Cancer Institute (NCI) over the number of entries that report a grant number of the National Institute of Health (NIH) of which the NCI is part. This ratio is an indication of the Cancer-funding from this Institute in the United States. It can be seen that the the ratio has been relatively constant at around 20% from 1980.

The second hypothesis is related to the funding: *more funding is available for Cancer research and therefore the proportion of publications has risen*. MEDLINE has a field for Grant Number [GR], which states the grant and the funder, for example: “*GR—R21 CA194661/CA/NCI NIH HHS/United States*”. The National Cancer Institute (NCI) (https://www.cancer.gov/) is part of the National Institute of Health (NIH) (https://www.nih.gov/), which in turn is which is one of 11 agencies that compose the Department of Health and Human Services (HHS) (https://www.hhs.gov/) of the USA. The NIH is a major funder of research with more than 2 million entries that list NIH in the grant number field. Thus, PubMed was queried for the entries with grant numbers that contained NIH or NCI, this last one is a subset of NIH. The number of grants is rather small from 1950 until 1980 when a rise of numbers is present. From this date, the ratio of NCI grants to the NIH is relatively constant at around 20% suggesting, that as a proportion of its funded research, the NIH has not changed from the 1980s (Fig 5).

**Fig 6:**
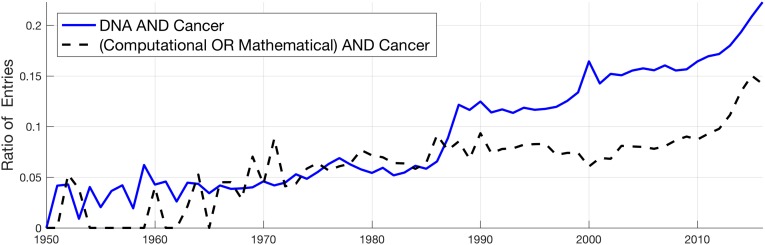
Ratios of all entries with the terms DNA and (Computational OR Mathematical), with and without cancer-related keywords as an indication of the impact that advances in these areas have had in cancer research. The ratio of Cancer has increased since the 1950s, with a particular surge in the mid 1980s for DNA.

The last hypothesis to be tested was the following: *scientific advances have had a higher impact in Cancer than in other diseases*. To test this hypothesis, it was necessary to select the relevant scientific advances with a relevant keyword through which to mine them in PubMed. The first scientific advance selected was the advent of genetic-related research. PubMed was thus queried for all publications that contained the keyword *DNA*. The output was then separated into two groups, those entries related to *Cancer* as defined by all the keywords previously described, and those that did not and presented as ratios of the total (solid line in Fig 6). The figure shows that the proportion of Cancer-related entries rises from around 5% in the early 1950s to nearly 30% in 2016, with a noticeable jump in the mid 1980s.

The increase in this test seems to confirm the hypotheses that genomic advances have had a higher impact in Cancer than in other diseases, and consequently, at least one of the reasons behind the general increase of Cancer-related entries in PubMed. Whilst for Cancer genomic-related advances include the discovery of oncogenes (i.e. *Src* [54–56]), tumour suppressor genes (i.e. *TP53* [57,58]) and mutations that confer well-known higher risks of cancer, e.g. *BRCA1*, *BRCA2* [59,60], the advances have not materialised so clearly for other conditions, like cardiovascular disease. It has been reported that few genome-wide studies have revealed “variants that, on their own, boost the risk of cardiovascular disease” [13]. Other trials showed that genetic tests did not show improved patient outcomes when observing patient’s genetic variants and dosing regimens of the anticoagulant drug warfarin [61]. As such, Cancer-related predictive genetic tests can be now performed, for instance, by the National Health Service in the UK [62].

A second scientific advance, which was tested was on scientific areas that may seem initially unrelated to Life Sciences, namely, Computing and Mathematics. To test these, PubMed was queried for the keywords (*Computational* OR *Mathematical*). The output was separated again in two groups, those that were Cancer related and those that did not include the previously defined keywords and the ratio was calculated (dashed line in Fig 6). A similar trend as that of DNA was found for the terms computational and mathematical; the proportion of Cancer-related entries has grown, before the 1970 it was zero in some years, then around 6–8% in the period 1970–1990, and has had a significant increase to around 14%.

The impact of mathematics and computing on Life Sciences has been identified in several areas. Recently, the newly appointed director of the US National Institute of Mental Health (NIMH) declared “To appreciate and exploit that complexity (of the brain) we need to integrate everything we know, from molecular biology to behaviour, into the models of how the brain works. That requires serious math.” [63]. There have been numerous publications in which different Cancer-related aspects have been investigated with mathematical and computational modelling, for instance: tumour vascular organisation [64], early-stage cancer interactions [65], tumour-induced angiogenesis [66,67], drug-transport in tumours [68,69], progression and aetiology of leukaemia [70,71], patient-specific drug treatments [72,73]. Some authors have stated that research related to Cancer has moved from solely experimental work to a combination with mathematical and computational modelling [74,75].

## Conclusion

This work developed a series of data mining tools as files of the software platform Matlab, which are available in S1 File. The tools were used to investigate the presence of Cancer-related entries in PubMed. It was found that Cancer-related entries have grown substantially from 6% in the 1950 to 16% in 2016. The hypothesis that that scientific advances in the areas of Genetics, Computing and Mathematics had a stronger influence in Cancer than other areas was supported by the results, which indicated an increase in Cancer-related PubMed entries as a ratio of the total. A particular change in the trends was noticed in the mid 1980s, and could be related to a series of developments in genetics in that time: Genome sequencing [76], development of Polymerase Chain Reaction (PCR) [77] or gene mapping of a human disease [78]. However, a finer mining is necessary to correlate individual advances like these ones with its impact on Cancer-related research.

Other factors could also influence the results. Cancer awareness is rising through efforts to improve literacy [79] promotion in schools [80] or training of health workers [81]. Patients diagnosed with Cancer are likely to survive a considerable period of time through which they will increase awareness. Diseases like atherosclerosis, on the other hand, develop ‘silently’ over decades before any symptoms occur [82].

This work has several limitations, of which the most important one is that only one database, albeit a very large one, was mined. There may be a substantial amount of non-reported research in commercial companies related to Cancer and other conditions that do not appear in PubMed. Second, the results depended on the presence or absence of keywords, some of these were examined in the section Terminology, but others were beyond the scope of this work, for instance, uterus/uterine. Third, there may be overlap of the results when one entry contains more than one keyword and thus the entries can be counted more than once.

Many other factors, which were not tested, could have an influence in the results presented. Besides funding from the NIH other sources of funding such as charities like *American Cancer Society*, *Cancer Research UK*, *National Foundation for Cancer Research* or *Worldwide Cancer Research* can have a significant impact in the research produced. The funding of pharmaceutical companies interested in developing cancer treatments may also be significant and have an impact on the research output, especially when compared against Malaria or other neglected diseases.

Cancer-related research has grown considerably. PubMed contains millions of publications, which describe techniques for diagnosis, management and treatment of Cancer. The oncologist Siddhartha Mukherjee wrote a comprehensive and captivating book titled “The Emperor of All Maladies, A Biography of Cancer” [83], which, interestingly, is not indexed in PubMed. At the beginning, within the Author’s Note, Mukherjee states that his ultimate aim is to raise the questions “*Is cancer’s end conceivable in the future*? *Is it possible to eradicate this disease from our bodies and societies forever*?” At this moment, the “Emperor” is the most prevalent disease in Europe [84], causes millions of deaths worldwide and is dominating the entries in PubMed. Whether, through the combined work of all authors behind those millions of publications, the Emperor can be ultimately contained and eradicated remains to be seen.

## Supporting information

S1 File DataMiningCode_PLOSONE_CancerInPubMed.zip. This compressed file contains the Matlab code that was used to query PubMed and to generate the figures of the paper.
(ZIP)
